# Tumor Stage-Based Gross Tumor Volume of Resectable Esophageal Squamous Cell Carcinoma Measured on CT: Association With Early Recurrence After Esophagectomy

**DOI:** 10.3389/fonc.2021.753797

**Published:** 2021-10-22

**Authors:** Yu-ping Wu, Sun Tang, Bang-guo Tan, Li-qin Yang, Fu-lin Lu, Tian-wu Chen, Jing Ou, Xiao-ming Zhang, Dan Gao, Ke-ying Li, Zi-yi Yu, Zhao Tang

**Affiliations:** Medical Imaging Key Laboratory of Sichuan Province, and Department of Radiology, Affiliated Hospital of North Sichuan Medical College, Nanchong, China

**Keywords:** esophagus, squamous cell carcinoma, recurrence, esophagectomy, tomography, X-ray computed

## Abstract

**Objective:**

To investigate relationship of tumor stage-based gross tumor volume (GTV) of esophageal squamous cell carcinoma (ESCC) measured on computed tomography (CT) with early recurrence (ER) after esophagectomy.

**Materials and Methods:**

Two hundred and four consecutive patients with resectable ESCC including 159 patients enrolled in the training cohort (TC) and 45 patients in validation cohort (VC) underwent contrast-enhanced CT less than 2 weeks before esophagectomy. GTV was retrospectively measured by multiplying sums of all tumor areas by section thickness. For the TC, univariate and multivariate analyses were performed to determine factors associated with ER. Mann-Whitney U test was conducted to compare GTV in patients with and without ER. Receiver operating characteristic (ROC) analysis was performed to determine if tumor stage-based GTV could predict ER. For the VC, unweighted Cohen’s Kappa tests were used to evaluate the performances of the previous ROC predictive models.

**Results:**

ER occurred in 63 of 159 patients (39.6%) in the TC. According to the univariate analysis, histologic differentiation, cT stage, cN stage, and GTV were associated with ER after esophagectomy (all *P*-values < 0.05). Multivariate analysis revealed that cT stage and GTV were independent risk factors with hazard ratios of 3.382 [95% confidence interval (CI): 1.533–7.459] and 1.222 (95% CI: 1.125–1.327), respectively (all *P*-values < 0.05). Mann-Whitney U tests showed that GTV could help differentiate between ESCC with and without ER in stages cT_1-4a_, cT_2_, and cT_3_ (all *P*-values < 0.001), and the ROC analysis demonstrated the corresponding cutoffs of 13.31, 17.22, and 17.83 cm^3^ with areas under the curve of more than 0.8, respectively. In the VC, the Kappa tests validated that the ROC predictive models had good performances for differentiating between ESCC with and without ER in stages cT_1-4a_, cT_2_, and cT_3_ with Cohen k of 0.696 (95% CI, 0.498–0.894), 0.733 (95% CI, 0.386–1.080), and 0.862 (95% CI, 0.603–1.121), respectively.

**Conclusion:**

GTV and cT stage can be independent risk factors of ER in ESCC after esophagectomy, and tumor stage-based GTV measured on CT can help predict ER.

## Introduction

Esophageal cancer is the seventh most common malignant tumor and the sixth leading cause of cancer-related deaths in the world (1, 2). Esophageal squamous cell carcinoma (ESCC) and adenocarcinoma are the two main histologic types, and in the high-risk areas, 90% of cases are ESCC (3, 4). Esophagectomy is considered the optimal treatment for resectable esophageal cancer (1, 5). But the long-term outcome remains poor, and even with radical resection, the 5-year postoperative survival rate does not exceed 30%, and postoperative recurrence is the main cause of treatment failure (6, 7). Some works have reported that the incidence of postoperative recurrence of esophageal cancer is the highest in the first year after esophagectomy with the incidence of more than 50%, and the median survival time after recurrence is less than 1 year (8–10). Hence, it is very important to predict the postoperative early recurrence (ER) of ESCC by integrating various factors and indicators.

Computed tomography (CT) plays a vital role in the management of esophageal cancer, such as diagnosis, treatment guidance, and follow-up (11, 12). With the continuous development of imaging technology, there are many alternative parameters that emerged, which are helpful for outcome prediction, and gross tumor volume (GTV) obtained on CT has been considered as one of these parameters (13). There are some researches about the relationship between GTV and the tumor stage, nodal disease, or treatment response based on imaging technologies to date. Lagarde et al. reported that the larger the tumor volume of esophageal cancer, the more likely lymph node metastasis and distant organ metastasis, and the greater possibility of incomplete surgical resection (14). Blom et al. reported that tumor volume of adenocarcinoma of the esophagus and gastroesophageal junction had an impact on tumor response to chemotherapy (15). Wu et al. suggested that GTV of resectable ESCC measured with MRI correlated well with T category and lymphatic metastasis (16). To the best of our knowledge, there are no reports to demonstrate if GTV of ESCC could predict the postoperative ER after esophagectomy. This study was devoted to investigating the factors associated with postoperative ER of resectable ESCC after esophagectomy, and the relationship of tumor stage-based GTV with ER.

## Materials and Methods

### Patients

This study was approved by the institutional ethics committee of our hospital, and each patient signed informed consent before participating in this study.

From March 2016 to June 2018, patients with ESCC proved by endoscopic biopsy were recruited into our study according to the following inclusion criteria: (a) patients did not receive any tumor-related treatments (e.g., chemotherapy or radiotherapy) before undergoing CT; (b) the tumors were resectable according to the National Comprehensive Cancer Network (NCCN) guidelines based on the CT manifestations (11); and (c) patients underwent esophagectomy with no residual disease at surgical margins, and were regularly followed up after surgery following the previous guidelines. There were 218 consecutive cases according to the inclusion criteria. The exclusion criteria were as follows: (a) patients had other thoracic surgery history (n = 6); (b) patients had other serious illnesses that made the patient cannot tolerate the surgery (n = 5); or (c) the quality of the images is poor (n = 3). The previous CT image quality was subjectively analyzed on a five-point scale (1, worst; 2, suboptimal; 3, adequate; 4, very good; 5, excellent) by two senior radiologists according to the image-quality scoring system (17). Therefore, 14 cases were excluded from our study, and our study ultimately involved 204 patients. All patients were randomly divided into a training cohort (TC, 159 patients) and a validation cohort (VC, 45 patients). The clinical, surgical, and pathological data in both cohorts were collected from the clinical database. All involved patients underwent thoracic contrast-enhanced CT examination within 2 weeks before the esophagectomy.

### Tumor Lesion Characteristics

According to the postoperative histopathology and American Joint Committee on Cancer (AJCC) staging system of esophageal cancer (18), the tumor anatomic location, the differentiation, T stage, N stage, and postoperative chemoradiation are listed in **Table 1**.

**Table 1:** Demographic and clinical information of the enrolled patients.

Variable	Training cohort	Validation cohort
Total no. of patients (NER : ER)	159 (96:63)	45 (25:18)
Sex, male:female	112:47	33:12
Age, median (range) in year	63 (41–78)	65 (41–79)
Postoperative therapy, yes:no	51:108	25:20
Differentiation		
Poor	75	17
Moderate	69	23
Well	15	5
Anatomical distribution		
Upper thoracic segment	17	5
Middle thoracic segment	114	30
Lower thoracic segment	28	10
T stage		
cT_1_	18	4
cT_2_	46	20
cT_3_	86	16
cT_4a_	9	5
N stage		
cN_0_	103	23
cN_1_	40	15
cN_2_	16	7
GTV, mean ± SD (cm^3^)	16.90 ± 10.80	21.20 ± 19.26

NER, non-early recurrence; ER, early recurrence; GTV, gross tumor volume; SD, standard deviation.

### Follow-Up and Definition of ER

During the follow-up period, all patients underwent postoperative medical and blood examinations and thoracic CT imaging or barium-swallow every 3–6 months in the first year after surgery. If recurrence was suspected based on the above examinations or patients had clinical symptoms associated with recurrence after surgery, patients underwent further examinations like cervical ultrasonography, thoracoabdominal CT, and endoscopic examination with biopsy. More selective investigations such as bone scintigraphy and cerebral CT were carried out based on specific symptomatology, clinical examination, and biochemical profile.

Based on the published literatures (9, 19, 20), we defined the recurrence within 12 months after surgery as ER. Subsequently, we divided our involved patients into the ER group and the non-ER group in the TC and VC. The patterns of the ER were classified into three types including local recurrence (at the site of the primary tumor or anastomotic and residual tissue recurrence), regional recurrence (regional lymph node recurrence), and distant recurrence (distant organ recurrence) according to the previous studies (6, 9).

### Contrast-Enhanced CT Scans

The CT data were obtained with enhanced 64-section multidetector computed tomography (MDCT) (LightSpeed VCT, GE Medical systems, USA). One hundred to 200 ml of water was given orally as negative contrast material before CT data acquisitions. All examinations were carried out in the supine position. After routine unenhanced CT scans, the contrast-enhanced CT data were obtained 25–30 s after the initiation of contrast agent (Omnipaque, Iohexol, GE Healthcare, USA) injection *via* a 20-G needle into an antecubital vein at a rate of 3.0 ml/s for a total of 70–100 ml tailored to body weight at the ratio of 1.5 ml/kg weight, followed by a 20 ml saline flush with a pump injector (Vistron CT Injection System, Medrad, USA). The CT scanning parameters were as follows: 120 kV of peak voltage, 200 mA of tube current (automatic exposure control employed), rotation time of 0.5 s, collimation of 64 × 0.6 mm, pitch of 0.9, slice thickness of 5 mm, and matrix of 512 × 512 mm. Examinations were performed during one breath-hold at full suspended inspiration for 10–15 s. The coverage of CT scan was from the neck to the middle of the left kidney. Subsequently, CT data were directly transferred to the General Electric Advantage Workstation 4.4 at the mediastinal window settings with window width of 400 HU and window level of 40 HU.

### Gross Tumor Volume Measurement

**Figure 1:**
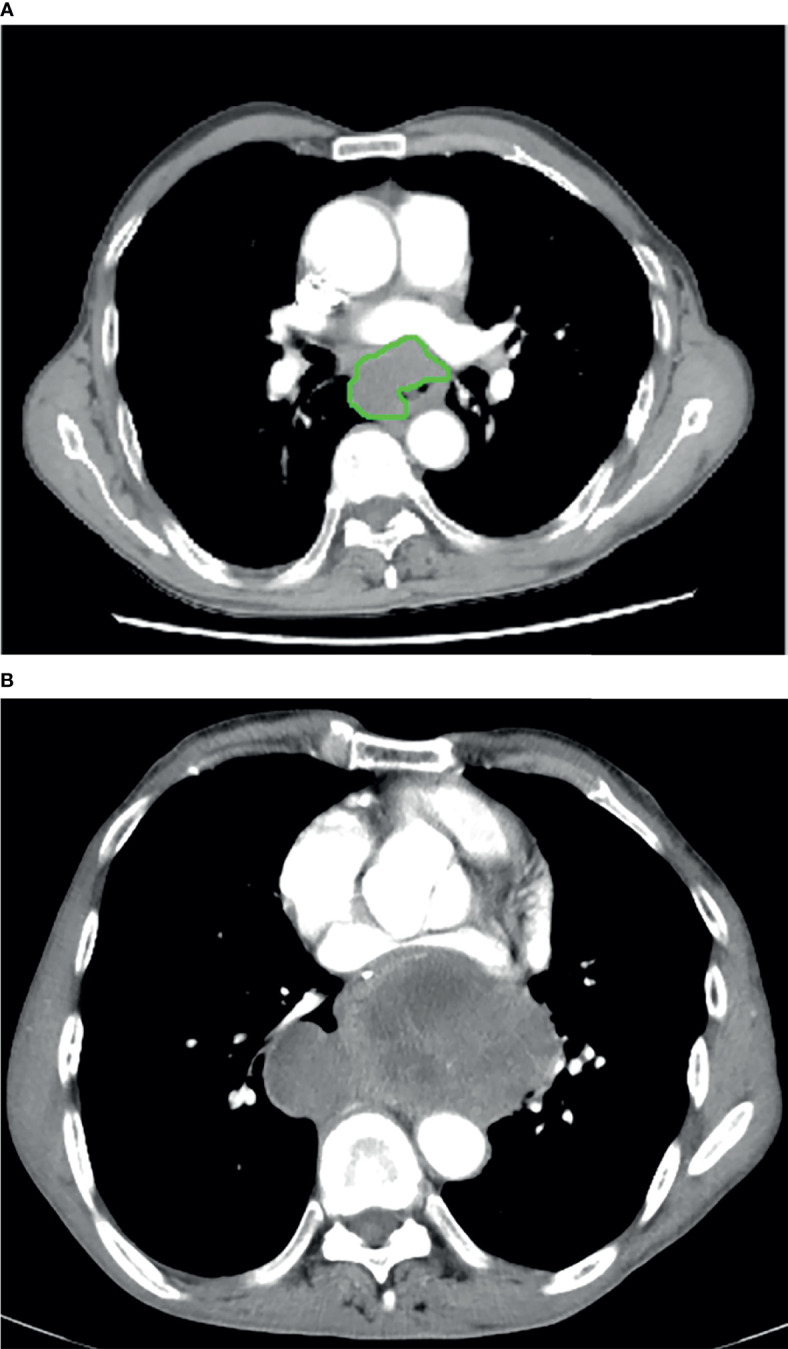
In a 61-year-old male with esophageal squamous cell carcinoma at cT_2_N_0_M_0_, the preoperative thoracic contrast-enhanced CT scans depict the gross tumor volume obtained by manual delineation along the margin of the abnormal esophageal wall **(A)** slice-by-slice, and the gross tumor volume is 58.66 cm^3^. Regional and local recurrence has been found 12 months after radical esophagectomy during the follow-up period **(B)**.

**Figure 2:**
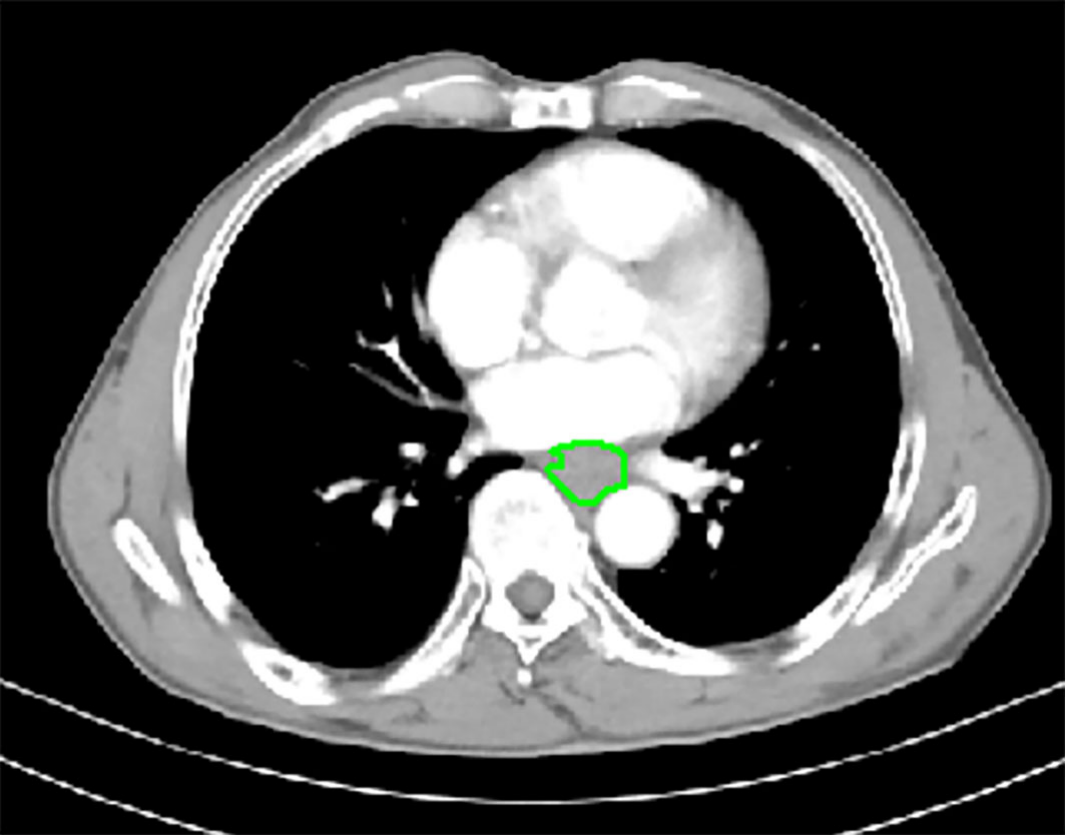
In a 66-year-old male with esophageal squamous cell carcinoma at cT_3_N_0_M_0_, the preoperative thoracic contrast-enhanced CT scans depict the gross tumor volume obtained by manual delineation along the margin of the abnormal esophageal wall slice-by-slice, and the gross tumor volume is 12.83 cm^3^. During the follow-up period, there was no recurrence as shown on follow-up CT after radical esophagectomy.

The GTV of resectable ESCC was measured on the abovementioned workstation, obtained by multiplying the sums of all tumor areas by the section thickness according to the method used in the published studies (21, 22). When the thickness of the esophageal wall was more than 5 mm on the axial contrast-enhanced CT scan, it was considered as the abnormal thickness caused by the tumor (23). The shape of the tumor was manually delineated along the margin of the abnormal esophageal wall (**Figures 1A**, **2**), and then the software automatically calculated the tumor area on each contiguous tumor section. Ultimately, the tumor areas were summed and subsequently multiplied by the layer thickness to obtain the GTV. In order to minimize measurement errors, we tried to avoid air and liquid within the esophageal lumen as much as possible when we sketched the tumor.

To ensure the accuracy of the tumor volume measurement, two experienced radiologists (Observer 1 with 3 years of radiology expertise, and Observer 2 with 5 years of experience in radiology) measured the GTV of all patients in the TC independently to verify the interobserver reproducibility. To verify the intraobserver reproducibility, the first radiologist remeasured the GTV of all patients 1 month later. Before the radiologists outlined the tumor to obtain the GTV, a professor of radiology with 23 years of experience in body radiology trained them how to draw the outlines of the tumor in 10 patients at random. All measurements were carried out without knowing the histologic results.

### Statistical Analysis

The IBM SPSS statistics software (version 25.0 for Windows; SPSS, Chicago, IL, USA) was used for the statistical analysis of data. A *P* value less than 0.05 was defined as a significant difference for all data. The reliability of repeated measurements of GTV was assessed *via* the intraclass correlation coefficient (ICC). ICCs less than 0.5, between 0.5 and 0.75, between 0.75 and 0.9, and greater than 0.90 are indicative of poor, moderate, good, and excellent reliability, respectively (24).

The continuous variables were expressed as mean ± standard deviation (SD). Categorical variables were shown as numbers and percentages. For the TC, univariate and multivariate analyses were performed to determine factors associated with ER. The Chi-square test or Fisher test was used to assess the univariate associations of possible factors with recurrence of resectable ESCC 1 year after the surgery. If the variables were statistically different at a *P* value less than 0.05 in the univariate analysis, they were enrolled in the multivariate analysis, which was performed by the binary logistic regression analysis to clarify the independent risk factors of the postoperative ER of resectable ESCC. The Mann-Whitney U test was used to compare GTV between patients with and without ER on the basis of different T stages. When a significant difference was proved by the previous Mann-Whitney U test, receiver operating characteristic (ROC) analyses were then carried out to determine if the cutoff values of GTV could be helpful to predict ER. Unweighted Cohen’s Kappa tests were conducted to evaluate the performances of the previous ROC predictive models to predict ER in the VC dataset independently. We used unweighted Cohen’s Kappa test according to the following rating scheme: less than 0.20, poor agreement; 0.21 to 0.40, fair agreement; 0.41 to 0.60, moderate agreement; 0.61 to 0.80, good agreement; and greater than 0.81, very good agreement (25).

## Results

### Patterns of ER of ESCC

In TC, ER occurred in 63 cases, and the rate of ER was 39.62% (63/159) while the remaining 96 patients did not have the recurrence. In detail, there were 4 (6.3%), 30 (47.6%), 6 (9.5%), and 23 (36.5%) patients who had local recurrence, regional recurrence, distant recurrence, and recurrence of two or more patterns 1 year after surgery, respectively. Of the 23 patients with recurrences of two or more patterns, there were 3 (13.0%) patients with local recurrence and regional recurrence; 8 (34.8%) patients had local and regional recurrence and distant recurrence in lung, liver, or bone; and 12 (52.2%) patients had regional and distant recurrence in lung, liver, or bone. In VC, ER occurred in 18 cases, and the rate of ER was 40.0% (18/45), among which regional recurrence was the most common recurrence pattern (11/18).

### Intra- and Interobserver Variability of GTV Measurements in the Training Cohort

For the initial measurement of the first observer, the mean GTV was 16.90 ± 10.8 cm^3^. To ensure the accuracy of GTV measurement, the intra- and interobserver ICC values of the GTV repeated measurements were 0.984 [95% confidence interval (95%CI), 0.968–0.992] and 0.978 (95%CI, 0.913–0.978), respectively, each with a *P*-value less than 0.001, indicating excellent repeatability of the GTV measurements in the TC. Thus, the first measurement of the first observer was reproducible and could be subsequently used for the further statistical analyses.

### Univariate Analysis of Correlation of Both Clinicopathological Factors and Tumor Stage-Based GTV With ER of ESCC in the Training Cohort

The correlation of both the clinicopathological characteristics and GTV with ER is summarized in **Table 2**. The histologic differentiation, cT stage, cN stage, and the GTV were associated with ER. In detail, ER was more common in poorly differentiated patients than in well-differentiated patients, patient with a higher cT stage was associated with a higher likelihood of ER, patient with a higher cN stage was more likely to be with ER, and the larger the tumor, the higher the incidence of ER (**Figure 1B**) (all *P*-values < 0.05). However, statistical analysis showed no significant association between ER and postoperative therapy with the *P*-value of 0.588, suggesting that not postoperative therapy but radical surgical resection could be a very effective treatment strategy for resectable ESCC.

**Table 2:** Univariate analysis of clinicopathological factors and gross tumor volume correlated with early recurrence in the training cohort.

Variable	Early recurrence		*P* value
	Yes (n = 63)	No (n = 96)	
Sex			0.825
Male	45 (71.4)	67 (69.8)	
Female	18 (28.6)	29 (30.2)	
Age			0.821
<63	31 (49.2)	49 (51.0)	
≥63	32 (50.8)	47 (49.0)	
Postoperative therapy			0.675
Yes	19 (30.2)	32 (33.3)	
No	44 (69.8)	64 (66.7)	
Differentiation			0.023
Poor	33 (52.4)	42 (43.8)	
Moderate	29 (46.0)	40 (41.7)	
Well	1 (1.6)	14 (14.5)	
Anatomical distribution			0.880
Upper thoracic segment	6 (9.52)	11 (11.5)	
Middle thoracic segment	45 (71.43)	69 (71.9)	
Lower thoracic segment	12 (19.05)	16 (16.6)	
T stage			<0.0001
cT_1_	1 (1.6)	17 (17.7)	
cT_2_	13 (20.6)	33 (34.4)	
cT_3_	41 (65.1)	45 (46.9)	
cT_4a_	8 (12.7)	1 (1.0)	
N stage			0.002
cN_0_	32 (50.8)	71 (74.0)	
cN_1_	19 (30.2)	21 (21.9)	
cN_2_	12 (19.0)	4 (4.2)	
Gross tumor volume (cm^3^)			<0.0001
<16.9	17 (27.0)	74 (77.1)	
≥16.9	46 (73.0)	22 (22.9)	

The numbers in the parentheses are percentages.

### Multivariate Analysis of ER in Resectable ESCC in the Training Cohort

Based on the above significant factors obtained by the univariate analysis, histologic differentiation, cT stage, cN stage, and GTV were selected as potential independent risk factors, and the binary logistic regression analysis was performed to determine the independent risk factors of ER. The logistic regression analysis showed that cT stage and GTV were independent risk factors for ER after radical resection of resectable ESCC (*P* = 0.003 and <0.0001, hazard ratio = 3.382 and 1.222, 95% CI of 1.533–7.459 and 1.125–1.327, respectively).

### Association of Tumor Stage-Based GTV With ER of Resectable ESCC in the Training Cohort

Associations of tumor stage-based GTV with ER of resectable ESCC after surgery were analyzed by the Mann-Whitney U test. Because most of the patients in our study were in the cT_2_ and cT_3_ category whereas numbers of patients in stages cT_1_ and cT_4a_ were small, we did not perform the Mann-Whitney U tests separately focusing on stages cT_1_ and cT_4a_ but on the stages cT_2_ and cT_3_ (**Table 3**). As depicted by the Mann-Whitney U tests, the GTV could help identify patients with ER in stages cT_1-4a_, cT_2_, cT_3_ (all *P*-values < 0.0001).

**Table 3:** Receiver operating characteristic analysis of tumor stage-based gross tumor volume for predicting early recurrence of esophageal squamous cell carcinoma in the training cohort.

T categories	Cutoff (cm^3^)	AUC	Sen (%)	Spe (%)	PPV (%)	NPV (%)	Acc (%)
cT_1-4a_	13.31	0.84	90.5	60.4	90.5	60.4	72.3
cT_2_	17.22	0.89	84.6	84.8	84.6	84.9	84.8
cT_3_	17.83	0.83	68.3	80.0	68.3	80.0	74.4

AUC, area under the receiver operating characteristic curve; Sen, sensitivity; Spe, specificity; PPV, positive predictive value; NPV, negative predictive value; Acc, accuracy.

### ROC Analysis of Tumor Stage-Based GTV to Discriminate ESCC Between Patients With and Without ER in the Training Cohort

**Figure 3:**
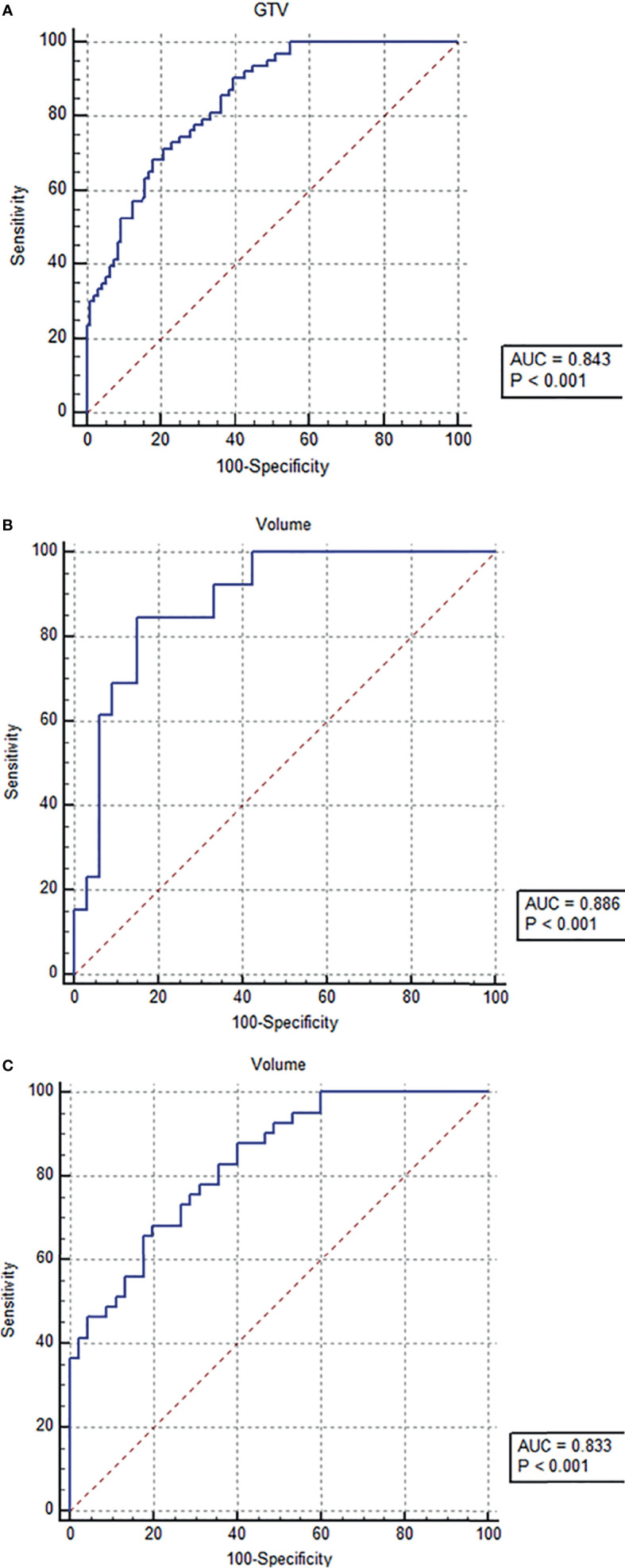
Receiver operating characteristic (ROC) analysis of tumor stage-based gross tumor volume (GTV) has been performed for predicting early recurrence of esophageal squamous cell carcinoma after esophagectomy, and the ROC curves show that the GTV can help predict early recurrence in tumor stages cT_1-4a_ **(A)**, cT_2_ **(B)**, and cT_3_ **(C)** with the cutoff values of 13.31, 17.22, and 17.83 cm^3^, respectively.

In order to demonstrate the accuracy of tumor stage-based GTV in predicting postoperative ER of resectable ESCC after esophagectomy, the ROC analysis was performed. According to the ROC analysis (**Figures 3A**–**C**), the GTV values were helpful for predicting ER of resectable ESCC after the radical surgery in patients in stages cT_1-4a_, cT_2_, and cT_3_ with the cutoff values of 13.31, 17.22, and 17.83 cm^3^, respectively. The area under the ROC curve (AUC), sensitivity, specificity, positive predictive value, negative predictive value, and accuracy of GTV for predicting ER of resectable ESCC are shown in **Table 3**.

### Unweighted Cohen’s Kappa Tests in the Validation Cohort for Validating the Performance of the ROC Predictive Models

In order to validate the extent of agreement in the diagnostic efficiency of the ROC models of tumor stage-based GTV for differentiating between ESCC with and without ER in stages cT_1-4a_, cT_2_, and cT_3_ with clinicopathological and follow-up results, unweighted Cohen’s Kappa tests were conducted in the VC according to the cutoff values obtained by ROC analyses of the TC. The tests revealed that the models obtained good agreements with clinicopathological and follow-up results in VC shown in **Table 4**.

**Table 4:** Unweighted Cohen’s kappa tests in the validation cohort for validating the performance of the predictive models.

T categories	Cohen K value	95% CI	*P*-value
cT_1-4a_	0.696	0.498–0.894	<0.0001
cT_2_	0.733	0.386–1.080	0.001
cT_3_	0.862	0.603–1.121	<0.0001

95% CI, 95% confidence interval.

## Discussion

The current study shows that the incidence of ER of resectable ESCC after radical esophagectomy could be associated with the cN stage, cT stage, degree of differentiation of the primary tumor, and GTV according to the univariate analysis. The GTV and cT stage are independent risk factors of ER in resectable ESCC. Except the GTV, the associations of the clinicopathological factors with ER of ESCC after radical esophagectomy were in accordance with published studies (19, 20, 26). In this study, for the first time, we found the association of the GTV with ER of ESCC after radical esophagectomy. In consideration for both independent risk factors (the cT stage and GTV), we further illustrated the feasibility of tumor stage-based GTV to predict ER of resectable ESCC after radical esophagectomy in TC.

As shown in our study, the GTV measured on CT could be an independent risk factor of ER in resectable ESCC. Tumor volume might be a comprehensive indicator reflecting the invasion length, tumor diameter, and the tumor depth of invasion. Hsu PK et al. found that the length of primary tumor is an important factor affecting recurrence of ESCC (27). And a previously published study showed that tumor length >6 cm was associated with distant recurrence (28). Both published papers suggest that the longer the tumor, the more likely the recurrence after esophagectomy. Gotohda et al. reported that the invasion of ESCC exceeded the submucosa, the lymph node metastasis rate increased significantly (29), and other published literatures showed the presence of metastatic lymph nodes was probably the main risk factor for the high incidence of recurrence in esophageal cancer (30, 31). We can presume that the invasion of ESCC exceeding the submucosa might be associated with ER of ESCC after esophagectomy. In addition, a study demonstrated that angiolymphatic invasion, submucosal invasion depth, and the largest diameter of invasion were independent predictors of early recurrence of ESCC (32). Based on the published studies on the relationship of early recurrence of ESCC with the invasion length, tumor diameter, and depth of invasion, we took all the possible risk factors of tumor size into consideration and chose the GTV as alternative parameter to explore the association of tumor size with ER after esophagectomy for the first time. Our study depicted that the GTV of ESCC was associated with ER after esophagectomy.

Our study showed that the GTV and tumor stage could be independent risk factors for ER of ESCC after esophagectomy. In consideration for both the cT stage and GTV, for the first time, we performed the stratification analysis of the GTV according to the cT stage to provide a new quantitative method for predicting ER of resectable ESCC after radical esophagectomy. Our study demonstrated that the stratification of GTV according to the tumor stage could obtain a good predictive performance on ER of ESCC. The ROC analyses showed that tumor-stage-based GTV measured on CT could predict ER of ESCC after radical esophagectomy with the AUC of more than 0.8.

Our study had several limitations. Firstly, this study enrolled a total of consecutive 204 patients in more than 2 years; the sample size was a little small. Especially, the numbers of patients in stage cT_1_ in the group of ER and patients in stage cT_4a_ in group of non-ER in both TC and VC are small, so we did not perform the ROC analyses separately. We will collect more patients in different cT stages in the future to confirm our findings. Secondly, GTV was obtained by manual delineation along the margin of the abnormal esophageal wall in our study. GTV measured by machine learning algorithm might be more reproducible, and the measurement time can be greatly reduced compared with the measurement in our study, but the repeatability of the GTV measurements in this cohort was still excellent, suggesting that our results were reproducible. Thirdly, because the routine CT scan thickness of thorax in our hospital was 5 mm rather than 1 mm, we performed this retrospective study by using 5 mm thick slices rather than 1 mm thick slices for tumor delineation. We will perform a prospective study by using 1 mm thick slices in the future to confirm our findings.

In conclusion, we found that GTV and cT stage can be independent risk factors of ER in resectable ESCC after esophagectomy for the first time. GTV measured on CT could predict ER of ESCC after radical esophagectomy, and the cT stage-based GTV has a better predictive performance on ER of resectable ESCC. We hope that our findings could help clinicians to generate new thoughts on the recurrence of ESCC after radical esophagectomy based on tumor stage-based GTV for timely appropriate intervention decision-making to improve the prognosis of ESCC.

## Data Availability Statement

The raw data supporting the conclusions of this article will be made available by the authors, without undue reservation.

## Ethics Statement

The studies involving human participants were reviewed and approved by the Affiliated Hospital of North Sichuan Medical College. The patients/participants provided their written informed consent to participate in this study.

## Author Contributions

T-WC, Y-PW, and ST conceived and designed the study. B-GT, L-QY, and F-LL collected the data. JO and DG analyzed the data and drafted the manuscript. All authors reviewed the manuscript, and T-WC revised the final manuscript. T-WC provided funding for the study. All authors contributed to the article and approved the submitted version.

## Funding

This work was supported by the National Natural Science Foundation of China (grant no. 81571645), the Sichuan Province Special Project for Youth Team of Science and Technology Innovation (grant no. 2015TD0029), and the Nanchong-University Cooperative Research Project (grant no: 20SXQT0329).

## Conflict of Interest

The authors declare that the research was conducted in the absence of any commercial or financial relationships that could be construed as a potential conflict of interest.

## Publisher’s Note

All claims expressed in this article are solely those of the authors and do not necessarily represent those of their affiliated organizations, or those of the publisher, the editors and the reviewers. Any product that may be evaluated in this article, or claim that may be made by its manufacturer, is not guaranteed or endorsed by the publisher.
